# Severe acute respiratory coronavirus virus 2 (SARS-CoV-2) screening among symptom-free healthcare workers

**DOI:** 10.1017/ice.2021.81

**Published:** 2021-03-12

**Authors:** Ryan T. Demmer, Angela K. Ulrich, Talia D. Wiggen, Ali Strickland, Brianna M. Naumchik, Shalini Kulasingam, Steven D. Stovitz, Clarisse Marotz, Pedro Belda-Ferre, Greg Humphrey, Peter De Hoff, Louise Laurent, Susan Kline, Rob Knight

**Affiliations:** 1 Division of Epidemiology and Community Health, School of Public Health, University of Minnesota, Minneapolis, Minnesota; 2 Department of Epidemiology, Columbia University, New York, New York; 3 Division of Environmental Health Sciences, School of Public Health, University of Minnesota, Minneapolis, Minnesota; 4 Medical School, University of Minnesota, Minneapolis, Minnesota; 5 Department of Family Medicine and Community Health, Medical School, University of Minnesota, Minneapolis, Minnesota; 6 Department of Pediatrics, University of California San Diego, La Jolla, California; 7 Department of Obstetrics, Gynecology, and Reproductive Sciences, Sanford Consortium for Regenerative Medicine, University of California, San Diego, California; 8 Division of Infectious Diseases and International Medicine, Medical School, University of Minnesota, Minneapolis, Minnesota; 9 Department of Computer Science & Engineering, Jacobs School of Engineering, University of California San Diego, La Jolla, California; 10 Department of Bioengineering, University of California San Diego, La Jolla, California; 11 Center for Microbiome Innovation, University of California San Diego, La Jolla, California

## Abstract

Transmission of severe acute respiratory syndrome coronavirus-2 (SARS-CoV-2) is possible among symptom-free individuals. Patients are avoiding medically necessary healthcare visits for fear of becoming infected in the healthcare setting. We screened 489 symptom-free healthcare workers for SARS-CoV-2 and found no positive results, strongly suggesting that the prevalence of SARS-CoV-2 was <1%.

Current evidence suggests that approximately half of severe acute respiratory syndrome coronavirus 2 (SARS-CoV-2) infections are due to transmission from symptom-free individuals.^1,2^ Healthcare workers (HCWs) may have an increased risk of SARS-CoV-2 infection, although it is also possible that the risk of infection among HCWs might be similar to community risk, as was recently reported in New York.^3^

Currently, data on the point prevalence of infection among symptom-free HCWs are limited. Given the evidence that patients are avoiding medically necessary healthcare visits for fear of becoming infected, prevalence estimates in HCWs can inform the potential risk that a patient might encounter an infected symptom-free HCW.^4^

To address this question, we screened symptom-free HCWs for SARS-CoV-2. Additionally, to preserve personal protective equipment (PPE), we implemented a protocol for self-collection of nasopharyngeal swabs (NPSs) and surveyed participants about their perceived quality of a self-collected versus provider-collected NPSs.

## Methods

**Fig. 1.**
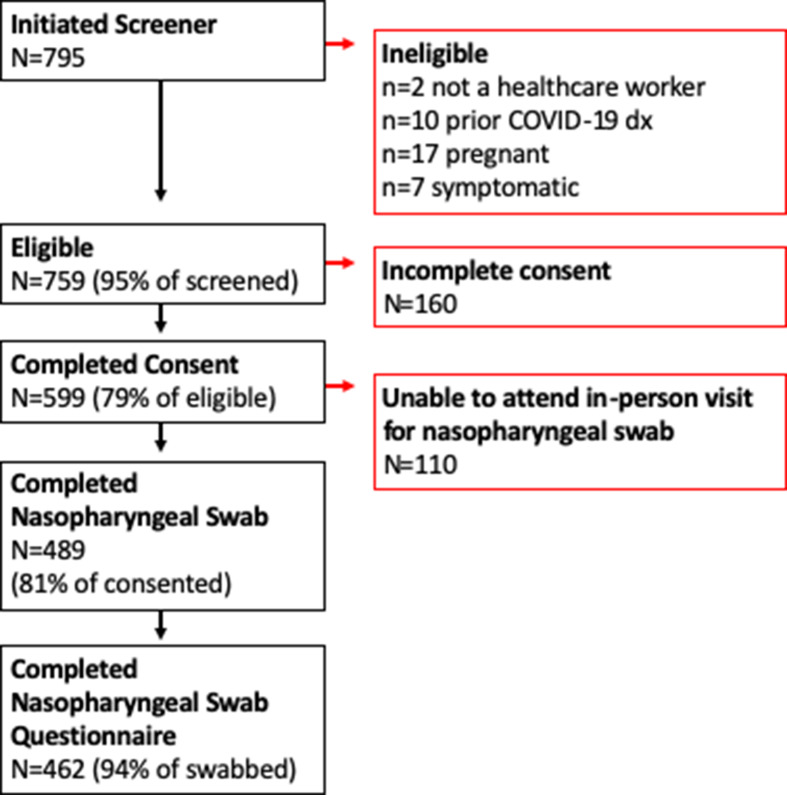
Participant flow diagram.

A convenience sample of individuals working in Minnesota healthcare facilities located in the Minneapolis–St Paul metropolitan area were enrolled. Participants were identified via social media advertisements and enrolled from April 20 to June 24, 2020. We applied the following eligibility criteria: (1) employed or volunteering in a healthcare facility; (2) free of fever, chills, anosmia, pharyngitis, recently developed persistent cough, nasal congestion suspected to be unrelated to season allergies; (3) aged 18–80 years; and (4) not pregnant. In total, 489 participants provided self-collected NPSs (Fig. 1). The study was approved by the University of Minnesota Institutional Review Board. All participants provided informed consent.

Participants advanced a nylon flocked-tip NPS through the nasal passage (bilaterally) 7.6–10 cm (3–4 inches) into the nasopharynx and swirled the swab 360° for 5 seconds. The swab tip was preserved in 3 mL 95% ethanol, immediately placed on an ice bath, and transferred to a −80°C freezer. Samples were shipped overnight on dry ice to University of California–San Diego.

Samples were processed within 48 hours of receipt at UC San Diego. Total nucleic acid was extracted from the swab heads using the MagMAX Microbiome Ultra Nucleic Acid Isolation Kit (no. A42357, Thermo Fisher Scientific, Waltham, MA) and eluted in 100 µL nuclease-free H_2_O. SARS-CoV-2 screening was performed using the one-step Applied Biosystems TaqPath COVID-19 Combo Kit (no. A47814, Applied Biosystems, Waltham, MA) following the manufacture’s protocol with the following exceptions. The reaction volume was scaled down to 3 µL with proportional reagent scaling and replacement of ∼94% of the water with participant RNA. Additionally, the MS2-phage spike-in control was diluted 160-fold to improve sensitivity through reducing competition for reagent material within the multiplex RT-qPCR reaction. Samples were prepared in 384-well reaction plates using a mosquito HV Robotic Liquid Handler (SPT Labtech, Melbourn, UK) and a mosquito X1 (HV) Robotic Liquid Handler (SPT Labtech). The RT-qPCR was analyzed in a QuantStudio5 qPCR instrument (Thermo Fisher Scientific). Positive controls for each SARS-CoV-2 target amplified as expected, as well as all MS2 sample controls. None of the negative controls amplified.

Prior to swabbing, participants completed online surveys. After the NPS procedure, participants were queried about their perception of the swabbing procedure relative to NPS they have performed on patients. They reported their level of discomfort with the self-swab on a scale of 1 (no discomfort) to 10 (the most discomfort they have ever experienced), and their likelihood of repeating a self-collected NPS for clinical or research purposes.

Descriptive characteristics are reported as mean (±SD) for continuous variables and number (%) for categorical variables. We used bivariate analyses, *t* tests, and χ^2^ tests to assess statistical significance. We had >95% power to detect at least 1 positive test if the true underlying prevalence of SARS-CoV2 was ≥1%.

## Results

Among 489 participants enrolled, the mean age was 41 (±11) years and 80% were female. All participants worked in facilities located in the 7-county Minneapolis–St Paul metropolitan area. The average number of people living with participants was 2 (SD±1.4) and 12% reported living alone. The average number of children living with participants was 0.9 (±1.1), and 50% reported having at least 1 child at home.

The average time between NPS collection and laboratory testing was 36 (±18) days (range, 2–68). SARS-CoV-2 was not detected in any sample.

In the 14 days prior to enrollment, 40% of participants reported a known COVID-19 exposure. This proportion varied by venue (*P* < .0001) and role (*P* < .01) (Table 1). PPE use was high with only 1.4% of participants reporting no PPE use and this occurred among individuals without patient contact.

**Table 1.** General Characteristics of 488 Minnesota Healthcare Workers^a^ According to COVID-19 Exposures Within 14 Days Preceding Enrollment^b^

Variable	All (N=488), No. (%)^c^	Known or Suspected COVID-19 Exposure, No (%)^d^		*P* Value
		Yes N=194 (40%)	No N=292 (60%)	
Age, y (SD)	41±11	38±0.7	42±0.67	<.0001
Sex, female	411 (84)	151 (77)	258 (88)	<.01
**Race**				.22
White	442 (90)	174 (90)	267 (92)	
Black	7 (2)	2 (1)	5 (2)	
Hispanic	9 (2)	4 (2)	5 (2)	
Other	30 (6)	14 (7)	15 (4)	
**Role**				<.01
Physician	70 (14)	23 (12)	47 (16)	
Nurse practitioner	37 (8)	14 (7)	23 (8)	
Physician assistant	19 (4)	8 (4)	11 (4)	
Nurse	251 (51)	111 (57)	139 (47)	
Paramedic/EMT	14 (3)	12 (6)	2 (1)	
Other	97 (18)	26 (14)	70 (24)	
**Setting**				<.0001
Emergency department	74 (15)	55 (28)	19 (6.5)	
Inpatient ICU	93 (19)	58 (30)	34 (12)	
Inpatient, other	137 (28)	50 (26)	87 (30)	
Ambulatory clinic	101 (21)	11 (6)	90 (31)	
Emergency transport vehicle	9 (2)	7 (4)	2 (0.5)	
Other	74 (15)	13 (6)	60 (20)	
**PPE use last 14 d**				
N95 respirator	159 (33)	107 (55)	52 (18)	<.0001
Surgical mask	347 (71)	117 (60)	229 (78)	<.0001
N95+surgical mask	193 (40)	112 (58)	79 (27)	<.0001
Face shield	87 (18)	38 (20)	49 (17)	.59
PAPR	18 (4)	14 (7)	4 (1)	<.01
None	7 (1.4)	0 (0)	7 (2.4)	.09

Note. SD, standard deviation; SE, standard error; EMT, emergency medical technician; ICU, intensive care unit; PPE, personal protective equipment; PAPR, powered air-purifying respirator.

a 489 received an NPS, but survey data were not available for 1 participant who did not complete the questionnaire and was excluded from the table.

b Enrollment occurred between April 20 and June 24, 2020.

c Data presented as mean±SD.

d Data presented as mean±SE.

The mean score for discomfort related to the self-collected NPS was 4.5 (±2.0; range, 1–10). Among the 287 participants (62%) who reported performing an NPS on a patient, 89% indicated that their self-swabbing depth was greater than or equal to the depth of prior patient swabs, and 95% reported that their self-swab was greater than or equal to the duration of previous patient swabs. More than 95% of participants reported a willingness to repeat a self-collected NPS for either clinical or research purposes; 24% preferred a provider-collected swab, 57% preferred self-collection, and 19% reported no preference.

## Discussion

The major finding in this study was the lack of any SARS-CoV-2–positive individuals among a convenience sample of symptom-free HCWs. Based on our power calculations, this strongly suggests that the point prevalence of SARS-CoV-2 in our study sample of symptom-free HCWs was <1%. This finding is consistent with results in the US population and in Minnesota during the period these samples were collected. National seroprevalence estimates reported by the Centers for Disease Control ranged from 1% to 7%, and the estimate from Minnesota during the period from April 20 to May 12, 2020, was 2.2%.^5^ Low SARS-CoV-2 point prevalence in HCWs, despite increased relative risk for infection compared to the general population,^6^ is plausible because HCWs are prioritized to receive PPE and are trained in infection control. Our findings from the self-collected NPS survey suggest that the self-collection of NPS was acceptable to HCWs and that the collections were properly done.

The sensitivity of our screening tests might have been low due to the use of self-collected NPSs, although recent studies report self-collection protocols to have acceptable sensitivity.^7,8^ Tests among symptom-free individuals could also have reduced sensitivity; however, prior studies in asymptomatic pregnant women^9^ and residents of long-term care facilities^10^ have detected high SARS-CoV-2 prevalence. More research is necessary to validate PCR tests among symptom-free individuals, although preliminary findings from the SalivaDirect test, which has Federal Drug Administration emergency use authorization, are encouraging (https://www.fda.gov/media/141194/download). Because we used a convenience sample, our sample is not representative of all HCWs in Minnesota, nor is it representative of what future SARS-CoV-2 prevalence estimates might be among symptom-free HCWs in settings with high community prevalence. Our sample was largely comprised of individuals working in acute-care settings, and representation from long-term care facilities, where infection risk has been notably higher in Minnesota, was limited.

Our results suggest that even though the HCWs are very likely to be at increased relative risk for infection compared to the general population,^6^ the point prevalence of SARS-CoV-2 infection was low in symptom-free Minnesota HCWs. If true, the probability of encountering an infected symptom-free HCWs during a medically necessary healthcare visit is likely low when community point prevalence is low. Self-collected NP swabs are acceptable to participants. Ongoing monitoring of infection in HCWs will be important as the pandemic progresses and community transmission rises across the country.
